# Metabolic Profiling of VOCs Emitted by Bacteria Isolated from Pressure Ulcers and Treated with Different Concentrations of Bio-AgNPs

**DOI:** 10.3390/ijms22094696

**Published:** 2021-04-29

**Authors:** Fernanda Monedeiro, Viorica Railean-Plugaru, Maciej Monedeiro-Milanowski, Paweł Pomastowski, Bogusław Buszewski

**Affiliations:** 1 Interdisciplinary Centre of Modern Technologies, Nicolaus Copernicus University in Toruń, 4 Wileńska St., 87-100 Toruń, Poland; fmonedeiro@gmail.com (F.M.); rviorela@yahoo.com (V.R.-P.); milanowski.maciej@gmail.com (M.M.-M.); p.pomastowski@umk.pl (P.P.); 2 Department of Environmental Chemistry and Bioanalytics, Faculty of Chemistry, Nicolaus Copernicus University in Toruń, 7 Gagarina St., 87-100 Toruń, Poland

**Keywords:** silver nanoparticles, VOCs, bacteria, pressure ulcers, GC–MS, pathway

## Abstract

Considering the advent of antibiotic resistance, the study of bacterial metabolic behavior stimulated by novel antimicrobial agents becomes a relevant tool to elucidate involved adaptive pathways. Profiling of volatile metabolites was performed to monitor alterations of bacterial metabolism induced by biosynthesized silver nanoparticles (bio-AgNPs). *Escherichia coli*, *Enterococcus faecalis*, *Klebsiella pneumoniae* and *Proteus mirabilis* were isolated from pressure ulcers, and their cultures were prepared in the presence/absence of bio-AgNPs at 12.5, 25 and 50 µg mL^−1^. Headspace solid phase microextraction associated to gas chromatography–mass spectrometry was the employed analytical platform. At the lower concentration level, the agent promoted positive modulation of products of fermentation routes and bioactive volatiles, indicating an attempt of bacteria to adapt to an ongoing suppression of cellular respiration. Augmented response of aldehydes and other possible products of lipid oxidative cleavage was noticed for increasing levels of bio-AgNPs. The greatest concentration of agent caused a reduction of 44 to 80% in the variety of compounds found in the control samples. Pathway analysis indicated overall inhibition of amino acids and fatty acids routes. The present assessment may provide a deeper understanding of molecular mechanisms of bio-AgNPs and how the metabolic response of bacteria is untangled.

## 1. Introduction

The continuous emergence of drug-resistant pathogens brings up the need to develop new antimicrobial products which can supply enhanced performance for the treatment of infections and asepsis [1]. Chronical wounds represent a burden to healthcare facilities and are a cause of morbidity among affected patients. Hosted microbes develop complex interactions between themselves and the environment, making healing assessment imprecise and precluding a definitive treatment protocol [2]. Precisely, the prevalence of pressure ulcers reaches around 38% of hospitalized individuals, and such wounds have been found harboring multidrug-resistant microorganisms. Among other factors, increasingly common out-of-hospital care can contribute to the propagation of these resistant strains [3].

With the advances in the field of nanoscience, nanoscale materials have been studied as potential antimicrobial agents due to their enhanced chemical and physical properties, including their greater surface area, which allows efficient interaction with microorganisms [4]. In this context, silver nanoparticles (AgNPs) remain as the most popular nanomaterial displaying documented activity against pathogens, appearing enrolled in other several biomedical applications [5]. AgNPs can be embedded in dressings, dental materials, medical devices and lotions for their functional usage in the clinical setting [4]. Due to the great relevance of AgNPs in the medical practice, a thorough investigation of their mechanism of action is still required, in order to infer their long-term efficiency and safety for practical purposes. In addition, knowledge concerning mechanisms of bacterial adaptation to nanoparticles remains limited, demanding a further examination of the potentially involved molecular processes [6].

The mechanism of action of silver nanoparticles against bacteria may involve the following phenomena: (i) nanoparticles adherence onto the surface of cell wall [7], leading to increased permeability and death [8]; (ii) formation of cell-wall inhibited due to the anchoring of silver to the cell membrane [8,9]; (iii) modulation of cellular signals and the rise of oxidative stress, increasing cell porosity [8]; (iv) inactivation of thiol groups from enzymes and proteins [8,10]; (v) prevention of DNA replication, cell division and respiratory chain processes due to reaction of AgNPs with DNA molecules [8,10,11]; and (vi) inhibition of cell growth by dephosphorylation of protein substrates in bacteria [12].

“Green” nanotechnology aims to implement sustainable synthesis protocols, which primarily involve reduced generation of waste and toxic derivatives, as well as the employment of renewable feedstock [13]. The use of environmentally safe methods for the preparation of functional nanomaterials is crucial for their practical application [14]. Microbe-assisted synthesis of nanoparticles fits to the concept of green chemistry, generating effective antimicrobial products which are possibly less toxic than those obtained through conventional chemical synthesis [15]. Besides that, nanoparticles can be combined to natural matrices to give rise to novel biocomposites, achieving a greater range of properties and applications [16].

Metabolomics encompass the systematic evaluation of a subset of metabolites produced by an organism, in order to be correlated with molecular mechanisms of interest [17]. In this case, metabolomics configures as a distinguished approach to study the impact of antimicrobial materials on biologic systems. Bacteria produce a large spectrum of volatile metabolites related to essential pathways and possess valuable biochemical meaning [18]. Such volatile organic compounds (VOCs) can be conveniently assessed through sampling of culture’s headspace [19]. Acquired VOC profiles can reflect metabolic disturbances suffered by bacteria and be a useful tool to identify novel mechanisms involved in the antibacterial activity of AgNPs, also enabling us to observe how metabolic responses vary among species. In addition, VOC trends can be correlated with physiological parameters (e.g., growth profile and possibly active pathways) and serve as indicators of agent effectuality. Such an experimental approach was previously explored for the investigation of bacterial volatiles generated by salivary bacteria [20,21]. The headspace of bacteria incubated with AgNPs at a fixed concentration was assessed at different time points, in order to obtain a qualitative snapshot of the current VOC emissions—the results suggested bacteria inhibition due to noticed alterations in the production of metabolic markers [20]. The fold-change of bacterial VOCs after AgNPs-treatment was correlated with paired alterations in protein profiles; the observed relationships were modeled to predict the effectiveness of the interaction bacteria-agent [21].

*Proteus mirabilis*, *Enterococcus faecalis*, *Klebsiella pneumoniae* and *Escherichia coli* have been reported as the most commonly identified microorganisms in pressure ulcer wounds [22,23]. Pressure ulcer isolates from *Proteus* and *Klebsiella* species have demonstrated multidrug resistance [24]. In parallel, *E. faecalis*, *K. pneumonia* and *E. coli* colonizing such types of wounds were noticed to be multi-resistant to different antibiotics [23,25]. Such a context justifies a particular interest in the stress-driven metabolic behavior of these bacterial species.

The aim of the present study was to map and elucidate alterations in the metabolism of *E. coli, E. faecalis, K. pneumoniae* and *P. mirabilis* isolated from pressure ulcers and cultivated in medium supplemented with biosynthesized silver nanoparticles (bio-AgNPs). For this purpose, volatile metabolites emitted by cultures were comprehensively analyzed, using HS-SPME/GC–MS (headspace solid-phase microextraction/gas chromatography coupled with mass spectrometry) as analytical platform. Information regarding metabolic response of bacteria to bio-AgNPs can contribute to the elucidation of agent’s action mechanism for specific pathogens. This approach can aid the development of novel methods for assessment of bacteria susceptibility and provide a deeper understanding on resistance mechanisms.

## 2. Results and Discussion

### 2.1. Bacteria Identification

**Figure 1:**
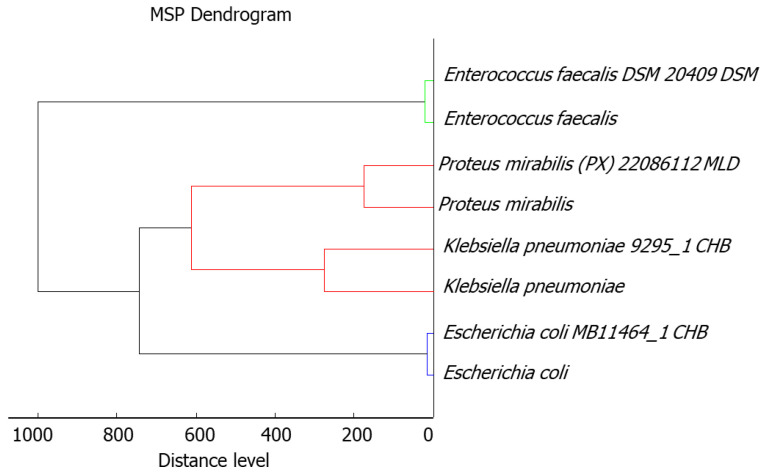
Phyloproteomic tree of the isolated bacterial strains, identified by MALDI Biotyper Compass platform.

Isolated bacteria strains were identified based on the Raw and Main Spectra (MSP), using a MALDI–TOF/TOF–MS (matrix-assisted laser desorption/ionization time-of-flight mass spectrometry) system. All of the isolated bacterial strains were identified with the high confident level, >1.999: 2.01 for *E. coli*, 2.00 for *E. faecalis*, and 2.03 for *K. pneumoniae* and *P. mirabilis.* The phyloproteomic relationships of the isolated bacterial strains revealed that these are closely related to the reference strain identified by MALDI Biotyper Compass platform (Figure 1).

### 2.2. Evaluation of Minimum Inhibitory Concentration (MIC)

In order to establish the metabolic profiling of VOCs emitted by bacteria isolated from pressure ulcers, firstly, the minimum inhibitory concentration (MIC) value was determined. The results indicate that all of the investigated bacterial strains (*E. coli*, *E. faecalis*, *K. pneumoniae* and *P. mirabilis*) presented similar susceptibility to bio-AgNPs; the MIC value for tested microorganisms was found to be 12.5 µg mL^−1^. Moreover, according to previous published results [15,26,27] it was observed that the antimicrobial potential of the bio-AgNPs is concentration-dependent; higher concentrations may induce not only the inhibition of bacterial growth but also bacterial death [15]. Therefore, for the investigation of bacterial VOC profiles, chosen concentrations of bio-AgNPs corresponded to MIC value of each strain and two concentration levels above this one (25 and 50 µg mL^−1^).

### 2.3. Unsupervised Inspection of Bacterial VOC Profiles

**Figure 2:**
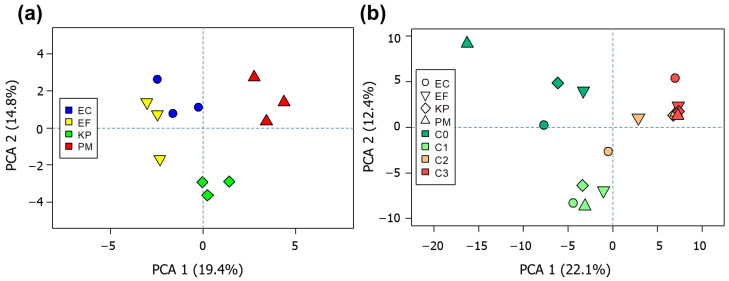
PCA score plots performed by using data regarding profiles obtained from (**a**) solely untreated cultures and (**b**) average of all performed assays (untreated + bio-AgNPs-treated cultures). C0 = assay without the addition of bio-AgNPs; C1, C2 and C3 = supplementation with bio-AgNPs at 12.5, 25 and 50 µg mL^−1^, respectively. EC = *E. coli*, EF = *E. faecalis*, KP = *K. pneumoniae*, PM = *P. mirabilis*.

The distribution of profiles and correlation between bacterial species were inferred by using principal component analysis (PCA). Considering solely the VOC profiles emitted by unstressed cultures (Figure 2a), the patterns of replicates belonging to the same species tend to be grouped together, indicating that VOC patterns are characteristic for each examined species. In addition, the set of volatile metabolites produced by *E. coli* and *E. faecalis* displayed more related patterns, while variances of *K. pneumoniae* and *P. mirabilis* were negatively correlated to the aforementioned species. When considering also VOC profiles from headspace of cultures supplemented with bio-AgNPs (Figure 2b), these appeared to be clustered according with the applied concentration of nanoparticles. This observation suggests that different levels of bio-AgNPs may induce metabolic shifts common among the studied species. Besides that, the patterns of variance in detected volatiles appeared to be more similar for the concentrations 25 and 50 µg mL^−1^, indicating the preservation of a similar basic metabolism at greater levels of the agent.

At this step, a preliminary inspection of the data’s general trend was aimed at observing if differentiation between species could be obtained spontaneously, considering the acquired global VOC profiles. In both cases, the first two principal components were able to describe only around 34% of the total variance observed within the data. This is possibly due to the fact that the studied bacteria share most of their VOC composition. This common fraction of the composition can be ascribed as belonging to bacterial fundamental metabolism at the given conditions. Such an explanation can be extended to the samples after bio-AgNPs treatment. In this sense, only a smaller portion of these profiles appear to be responsible for such simultaneous discriminations observed in the score plots.

**Figure 3:**
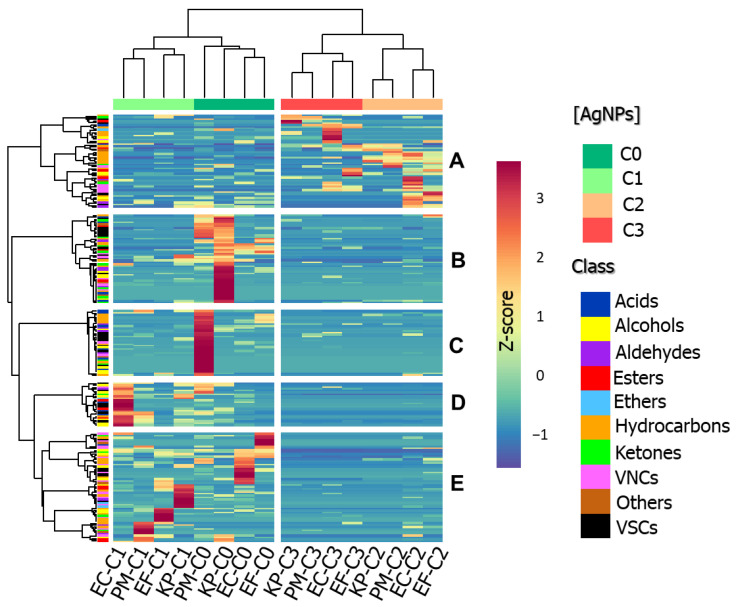
HCA associated with heatmap, displaying changes in VOCs’ intensities in studied volatile bacterial profiles and showing the assigned clusters (Pearson method). EC = *E. coli*, EF = *E. faecalis*, KP = *K. pneumoniae*, PM = *P. mirabilis*.

Hierarchical cluster analysis (HCA; Figure 3) displays average VOC profiles obtained for each assay ordered according to their similarity. Such approaches reinforce that profile composition tends to be governed by the added concentration of nanoparticles. With basis on the dendrograms presented at the top of the heatmap, the performed experiments can be divided into two main groups: unstressed cultures together with those supplemented with the lower concentration of bio-AgNPs (12.5 µg mL^−1^—C1) and the cultures treated with greater concentrations of the agent (25 and 50 µg mL^−1^—C2 and C3, respectively). These groups can be discriminated due to the differential response of compounds organized in the clusters A to E. The compounds contained in the cluster A, which are mostly hydrocarbons, presented increased responses in assays using greater concentrations of nanoparticles (C2 and C3). However, for clusters B, C, D and E, the opposite trend is observed.

### 2.4. Differences in VOC Profiles Composition

**Figure 4:**
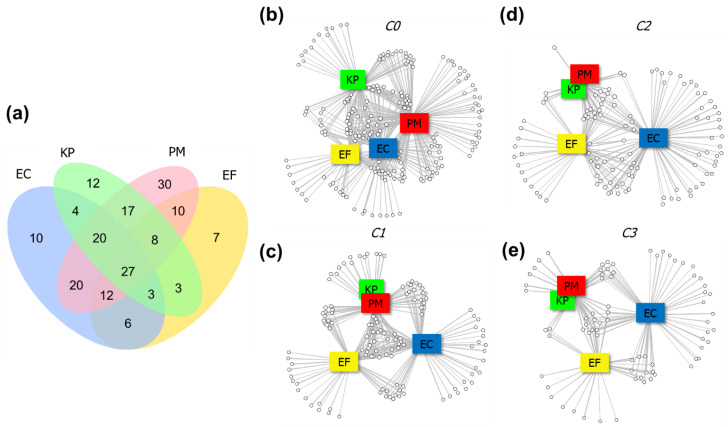
(**a**) Venn diagram displaying the number of compounds coincident between the studied untreated bacterial strains. Networks presenting the connections between the bacterial strains (squares) and respective emitted compounds (circles) for VOC profiles regarding (**b**) untreated cultures and those supplemented with bio-AgNPs at concentrations (**c**) 12.5, (**d**) 25 and (**e**) 50 µg mL^−1^. EC = *E. coli*, EF = *E. faecalis*, KP = *K. pneumoniae*, PM = *P. mirabilis*.

The Venn diagram exhibited in the Figure 4a refers to the incidence of compounds common among the investigated bacteria in untreated cultures. These values can indicate the degree of similarity between the original metabolism of the referred strains. In total, 189 different compounds were detected as being emitted by unstressed cultures; from these, 27 were coincident among all bacteria (approximately 14% of the total profile). The greatest profile similarity was observed for *E. coli* and *P. mirabilis*, which shared 20 VOCs. A total of 7, 10, 12 and 30 volatiles were found exclusively in the profiles of *E. faecalis*, *E. coli*, *K. pneumoniae* and *P. mirabilis*, respectively. In this sense, *E. faecalis* produces the less distinct pattern of volatiles, while *P. mirabilis*’ VOC profile is the most diverse among the studied strains. Few compounds were incident consistently in all replicates of a specific bacterium in the control cultures, namely 2-propanol and pentadecane for *E. coli*; acetoin, indazole and 3-phenylindole for *E. faecalis*; 3-methyl-1-butanol propanoate and 2-tetradecanone for *K. pneumoniae*; and methyl thiolacetate, 2-methylpropanoic acid, S-methyl thiobutyrate, 2-methylpyrrole-3-carbonitrile, octanoic acid, 2,4-dimethyldecane and methyl 4-tert-butyl-thiobenzoate for *P. mirabilis*.

The network analysis (Figure 4b–e) provides a graphical representation of the relationships existent between bacterial strains (rectangles) and their emitted VOCs (circles). These graphs can demonstrate the dynamics of alterations that occur in the set of bacterial metabolites coming from different species when increasing concentrations of bio-AgNPs are added to the medium. The addition of 12.5 µg mL^−1^ primarily induces a reduction in the variety of detected VOCs (139). Additionally, there is an increase in the occurrence of compounds generated by more than one bacterium: although the original set of VOCs had been reduced by 26%, the number of compounds shared among the three species increased (32), and exclusive volatiles are no longer observed in case of *P. mirabilis*. In contrast to this, for *E. coli* and *E. faecalis* the number of characteristic VOCs increased, indicating the activation of metabolic processes which were previously absent. The supplementation with 25 and 50 µg mL^−1^ of bio-AgNPs lead to progressive depletion in the number of produced bacterial metabolites. Overall, the VOC patterns of *K. pneumoniae* and *P. mirabilis* tend to converge, while *E. coli* develops the most distinct VOC profile.

**Figure 5:**
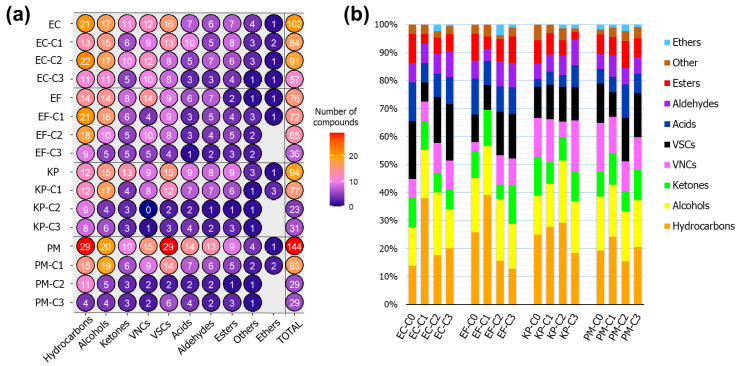
(**a**) Plot displaying the number of compounds detected in each of the performed experiments, according with functional group—the color scale refers to the range from lower (dark blue) to greater (red) incidence of VOCs; (**b**) bar plot presenting the proportion of VOCs detected referring to the respective chemical classes. EC = *E. coli*, EF = *E. faecalis*, KP = *K. pneumoniae*, PM = *P. mirabilis*.

Considering also the experiments involving bio-AgNPs treatment, a total of 220 compounds were detected. Figure 5a presents distribution of found VOCs according to their functional groups. Specifically, 102, 76, 94 and 144 different volatiles were found in the unstressed cultures of *E. coli*, *E. faecalis*, *K. pneumoniae* and *P. mirabilis*, respectively. Native VOC profiles from *E. coli*, *E. faecalis*, *K. pneumoniae* and *P. mirabilis* were mostly composed by hydrocarbons (14, 26, 25 and 19%), alcohols (14, 19, 14 and 10%), ketones (10, 10, 14 and 9%), volatile nitrogen compounds (VNCs) (7, 3, 14 and 18%), volatile sulfur compounds (VSCs) (21, 10, 11 and 14%), acids (14, 13, 3 and 5%), aldehydes (7, 6, 6 and 5%) and esters (10, 10, 8 and 7%), respectively. In terms of incidence, hydrocarbons, followed by alcohols and VSCs (or VNCs, in case of *E. faecalis*), were the predominant chemical classes among the obtained profiles. The differing numbers of VOCs detected in untreated cultures can be interpreted as being associated with the individual metabolic variability pertinent to each of the bacterial species, at the given environmental and nutritional conditions. Some steps of the pathways responsible for generating volatile metabolites may not be active in all of the studied bacteria. Moreover, originally displayed enzymatic activity can differ depending on the investigated organism.

When comparing the profiles generated by unstressed bacteria and those acquired using the greatest concentration of agent (50 µg mL^−1^), it is observed a reduction of 44, 52, 67 and 80% in the number of VOCs previously detected for *E. coli*, *E. faecalis*, *K. pneumoniae* and *P. mirabilis* (consecutively). The most abrupt decrease in volatiles number occurred for nanoparticles incremented at concentration of 25 µg mL^−1^ for *K. pneumoniae* and *P. mirabilis*, and 50 µg mL^−1^ for the remaining species. Such observations suggest that *E. coli* and *E. faecalis* display greater resistance to bio-AgNPs supplementation, in terms of preservation of their original metabolism. In general terms, the increase in the number of detected VOCs can be associated to the elicitation of molecular mechanisms induced by the introduced agent. These can possibly include the upregulation of certain pathways in order to compensate bacterial functions which were impaired, or to generate products with specific biological roles. VOCs generated after treatment can also be byproducts of chemical reactions with cell components promoted by nanoparticles. A reduction in the number of produced volatiles can be simply due to the inhibition of biochemical processes, or a consequence of metabolic shifts intending to adapt cell survival to the new environment.

Figure 5b expresses the changes in the proportion of compounds used in each of the performed assays, according to their respective chemical groups. In general, the contribution of hydrocarbons to the profiles from cultures processed with lower concentrations of bio-AgNPs tends to increase. This indicates that, at the MIC level, the occurred metabolic alterations encompassed the augmented production of this class of compounds. The biosynthesis of hydrocarbons is documented in several species of microorganisms, being mainly associated to the fatty acid metabolism. Precursor molecules of acetyl-CoA (or structural derivatives) are elongated with malonate units, the decarboxylation of the formed products can generate alkanes and alkenes (both straight and branched chain, depending on the employed starter unit) [18,28]. The composition of hydrocarbons emitted by microorganisms appears to be related to their physiological groups and the conditions provided for their growth [29]. Still, there is a lack of knowledge regarding the possible biological/physiological roles of biogenic hydrocarbons. It is believed that produced hydrocarbons may serve as source of carbon and energy for the cell. Above that, these compounds likely develop functions in regulation of membrane permeability [29].

Overall, the proportion of classes such as aldehydes (for all species), VNCs (in *E. coli*, *E. faecalis* and *K. pneumoniae*), VSCs (in *E. faecalis*, *K. pneumoniae* and *P. mirabilis*) and ethers (in *E. coli*, *E. faecalis* and *K. pneumoniae*) increased in profiles after bio-AgNPs treatment. Aldehydes are often reported as products of lipid peroxidation and could be generated due to cell membrane damage. The interaction of fatty acids with reactive oxygen species (ROS) produces radicals which tend to be stabilized through scissions, giving rise mainly to alkanes, alcohols and aldehydes [30]. For both *K. pneumoniae* and *P. mirabilis*, the percentage of acids raised with the addition of nanoparticles to the medium. Free fatty acids may occur due to some release from cell wall. However, the production of an increased variety of this class of compounds can be connected with alterations of plasma membrane composition. Previous research investigated alterations in plasma membrane composition of silver ions and silver nanoparticles: *Pseudomonas putida* modified the *trans*/*cis* ratio of unsaturated fatty acids in order to decrease membrane fluidity and counteract the wall permeability occasioned by silver ions [31]. A shift in the composition of lipids from bacterial membrane is also reported, in the sense that the portion of saturated fatty acids tends to increase, creating a “rigidifying effect” intended to stabilize the membrane after AgNPs incursion [32]. Considering that the metabolisms of *K. pneumoniae* and *P. mirabilis* appeared to be the most susceptible to the studied nanoparticles, an elevation in the number of fatty acids is possibly a consequence of cell wall degradation. Studies describing ROS-mediated mechanisms confirmed the occurrence of lipid peroxidation as a consequence of secondary damage during stress caused by the administration of bactericidal agents [33,34]. More specifically, ciprofloxacin was found promoting DNA and lipid oxidation in *Staphylococcus aureus*; lipid peroxidation was attested by the formation of malondialdehyde, a product of ROS interaction with unsaturated fatty acids and plasmatic marker of oxidative stress [35]. An investigation on *Lactobacillus helveticus* showed that fatty acid composition patterns were altered as a response to environmental stress conditions, possibly leading to ROS production [36].

### 2.5. Investigation of Discriminating Features

**Figure 6:**
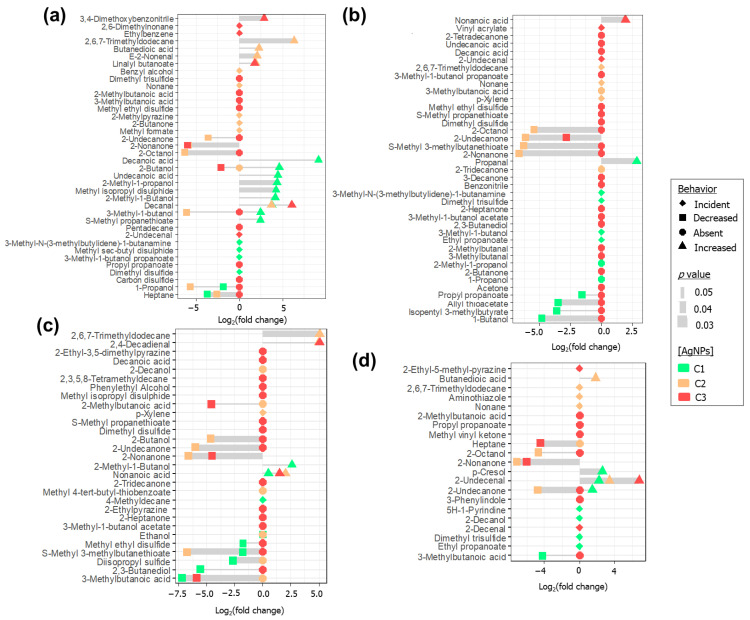
Plots showing alterations in VOCs’ response in treated samples in relation to untreated ones (expressed in terms of binary logarithm of fold change) for (**a**) *E. coli*, (**b**) *K. pneumoniae*, (**c**) *P. mirabilis* and (**d**) *E. faecalis*. Thickness of the edges are proportional to the significance of the alteration (based on p value). Four main compound behaviors are outlined: incident (diamonds) or absent (circles) only after bio-AgNPs treatment, and those that had increased (triangles) or decreased (squares) responses after the supplementation. C1, C2 and C3 = supplementation with bio-AgNPs at 12.5, 25 and 50 µg mL^−1^, respectively.

The plots in Figure 6 display the VOCs which presented statistically significant alterations in their responses (*p* < 0.05) after bio-AgNPs addition. Four types of alterations were verified: volatiles that had their areas increased or decreased when medium was treated with the nanoparticles, as well as those that became absent or incident only in the headspace of cultures supplemented with nanoparticles. For *E. coli*, *E. faecalis*, *K. pneumoniae* and *P. mirabilis*, the numbers of found altered features were 64, 36, 77 and 62, respectively. In general, the main observed effect was the absence of compounds previously found being emitted by the original cultures (disappearance of 23 VOCs in *E. coli*, 36 in *E. faecalis*, 56 in *K. pneumoniae* and 40 in *P. mirabilis*). However, such a phenomenon appears mostly associated with the processing of cultures with the greatest concentration of agent (C3). Significant changes related to the use of intermediary and lowest concentrations of bio-AgNPs (C2 and C1) refer mostly to the modulation in the response of the detected VOCs, as well as the incidence of new compounds. In this sense, C3 configures as a threshold level that induces irreversible modifications in the pattern of emitted bacterial metabolites, as a possible result of substantial metabolism inhibition.

General considerations can be drawn regarding the volatiles that became present only after supplementation with nanoparticles. C1 level led to the production of 3-methyl-1-butanol propanoate for *E. coli*; dimethyl disulfide for *E. coli*, *E. faecalis* and *K. pneumoniae*; 3-methyl-1-butanol for *K. pneumoniae*; ethyl propanoate for *K. pneumoniae* and *E. faecalis*; 4-methyldecane for *P. mirabilis*; and 2-decanol for *E. faecalis*. Dimethyl disulfide can have its origin in the degradation of methionine. The formed Strecker aldehyde (methional) can be converted into methanethiol, and such species can auto-oxidize to produce sulfides [37]. A previous study described the stimulation of antibiotic resistance in *E. coli* exposed to dimethyl disulfide, demonstrating that volatiles emitted by *Burkholderia ambifaria* strains can influence the development of other organisms [38]. Moreover, 3-methyl-1-butanol is probably derived from enzymatic conversion of leucine-derived keto acid by Ehrlich pathway. The α-keto acids produced during the processing of amino acids are transformed into the corresponding alcohols after action of the α-ketoacid decarboxylase and alcohol dehydrogenase enzymes [39,40,41]. These “fusel alcohols” can undergo esterification by activity of alcohol acyltransferase enzyme [42,43]. In this sense, esters such as 3-methyl-1-butanol propanoate and ethyl propanoate, may be sourced by this pathway. Among other alcohols, 3-methyl-1-butanol (isoamyl alcohol) proved to promote biofilm formation in strains of *S. aureus.* The alcohol treatment triggered the transcription of genes related to biofilm build-up and promotion of antibiotic resistance [44]. Specifically, this same concentration level also modulates positively a series of compounds. Decanoic acid, undecanoic acid (increased in *E. coli*) and nonanoic acid (increased in *P. mirabilis*) can be derived from stimulation of fatty acid synthesis. Indeed, a study showed that membrane disruption provoked by AgNPs triggered the biosynthesis of lipids in *E. coli*, possibly as a mechanism to recover membrane integrity [32]. Decanal and 2-undecenal (elevated in *E. coli* and *E. faecalis*, respectively) can be obtained through the β and α scission of oleic acid after its peroxidation in the position C8 [30]. Propanal (augmented in *K. pneumoniae*) can be generated during fermentation of monosaccharides, during dehydration of propane-1,2-diol [45,46]. Alternatively, mentioned saturated aldehydes could be obtained by the reduction of intermediaries from fatty acid elongation step [18]. The fermentation of glucose under conditions deprived of oxygen can produce acetoin as metabolite, which in its turn can be reduced to 2,3-butanediol. 2,3-Butanediol is dehydrated to 2-butanone, which may be reduced to 2-butanol—a VOC augmented in *E. coli*, possibly evidencing an upregulation of anaerobic sugar fermentation [47]. The increasing of compounds such as 2-methyl-1-propanol and 2-methyl-1-butanol (in *E. coli*, and the latter also in *P. mirabilis*) suggest enhancement of Ehrlich pathway. Moreover, *p*-cresol, found elevated in *E. faecalis*, can be obtained by fermentation of tyrosine and it is typically produced by *Clostridium difficile*, another Gram-positive bacterium [48]. *p*-Cresol has bacteriostatic properties—in a specific microbiota, its production can confer competitive advantages over other colonizing pathogens. In that way, release of *p*-cresol is related to bacteria mechanisms for enhancement of their pathogenesis and survival [49].

C2 resulted in the appearing of 2-methylpyrazine, 2-butanone and methyl formate for *E. coli*; nonane for *E. coli*, *K. pneumoniae* and *E. faecalis*; 2,6,7-trimethyldodecane for *K. pneumoniae* and *E. faecalis*; *p*-xylene for *K. pneumoniae* and *P. mirabilis*; ethanol for *P. mirabilis*; and butanedioic acid in *E. faecalis*. Pyrazine derivatives are possibly descendant from the biotransformation of tryptophan [18]. They are outlined as biologically active bacterial volatiles with antifungal and antimicrobial properties. Besides that, there is evidence on their antioxidant activity, a property beneficial for resistance to free radicals [50,51]. As described previously, 2-butanone is a product of pyruvate–diacetyl/acetoin pathway [47], evidencing a stimulation of alternative pathways for obtaining of energy. However, its reduction product (2-butanol) became absent at the present concentration level (C2), indicating that the conversion of 2-butanol to 2-butanone has been interrupted; this can be possibly related to a lack of reduced co-factors in the environment. Additionally, 2-butanone was found inducing bacterial motility in *Pseudomonas aeruginosa* [52]; therefore, this can be a VOC with biologically active response that suits to the stress experienced by bacteria in this circumstance. Facultative anaerobic bacteria such as those here studied can perform glucose fermentation. The process begins with the obtaining of phosphoenolpyruvate (PEP), which is then transformed into pyruvate and can be subsequently reduced to ethanol. PEP can also be transformed to oxaloacetate, which is enzymatically converted into succinate (butanedioic acid). Research on *Actinobacillus* sp. and *E. coli* showed that ratio of production of ethanol and butanedioic acid are dependent on the CO_2_ concentrations and used source of electrons [53]. As for 3-methyl-1-butanol, supplementation with ethanol at low concentrations in *Staphylococcus* cultures proved to enhance the formation of biofilm and engender the expression of biofilm-promoting genes, potentially increasing resistance and pathogen potential of bacteria [44,54].

Residual levels of silver ions are commonly present dissolved or adsorbed to the nanoparticles coating. Additionally, Ag^+^ can be generated during the oxidation of nanoparticles in aqueous environment under acidic conditions, therefore promoting the release of silver ions under aerobic conditions [55]. Ag^+^ released in the vicinity of the bacteria can lead to the disruption of cell wall and membrane, thus configuring as a AgNPs-associated biocide mechanism [56]. Observed elevated levels of succinate may be linked to the hampering of cellular respiration: silver ions were found inhibiting the oxidation of glucose and causing decrease in the levels of this intermediary [57]. Furthermore, Ag^+^ seemed to impair transport of ions in bacterial cell, resulting in an outflow of succinate. Such mechanism is possibly connected with the inhibition of NADP-linked glutamate dehydrogenase [58]. Specifically, at C2, an augmentation in the responses of the following VOCs was verified: 2,6,7-trimethyldodecane (in *E. coli* and *P. mirabilis*), butanedioic acid (in *E. coli* and *E. faecalis*) and 2-nonenal (in *E. coli*). 2-Nonenal may be yielded during the oxidative breakdown of palmitoleic acid [59]. Regarding the appearing of *p*-xylene, aromatic compounds are reported to be synthetized by the shikimate pathway—a biochemical route responsible for production of aromatic amino acids [60]. It was found that silver ions displace copper ions operating in enzymatic processes as those related to the shikimate pathway, including themselves in the active sites of involved enzymes [61]. Therefore, the enzymatic generation of differential aromatic products may be related to such imbalance. However, connected with this factor, unbound copper results in the deficit of cuproenzymes, which serve as cellular antioxidative defenses, thus allowing ROS to accumulate in the periplasmic space [61].

C3 caused the occurrence of only few volatiles, such as 2-undecenal (*E. coli* and *K. pneumoniae)* and 2-decenal (*E. faecalis*). Like 2-undecenal, 2-decenal can also be generated by the oxidation of lipids from plasma membrane, particularly through the α scission of oleic acid after interaction with ROS. At C2 and C3, continuous increasing of compounds such as decanal (for *E. coli*), nonanoic acid (for *P. mirabilis*) and 2-undecenal (for *E. faecalis*) was observed. Nonanoic acid, despite possibly being formed by fatty acids biosynthesis pathway, can also be released through α scission of oleic acid after oxidized in the position C-10. Besides the disappearing of several VOCs previously generated by the cultures, increasing concentrations of agent resulted in elevated responses of compounds potentially related to cell membrane damage. Indeed, plasma membrane is described as the main target of AgNPs. Further effects associated with this phenomenon were loss of potassium ions from the intracellular space and decreased production of energy (ATP) [62]. The profile of straight-chain saturated and mono-unsaturated fatty acids for both Gram-negative and Gram-positive bacteria appear to be rather similar, mainly consisting of palmitic (C16:0), oleic (C18:1), palmitoleic (C16:1) and stearic (C18:0) acids [63]. Due to this, the correlation made between some of the discriminating features as products of lipid oxidative cleavage appear to be reasonable, once these involve oleic and palmitoleic acids (principal unsaturated lipids of bacteria membrane) as main substrates.

A part of the aforementioned compounds has in common their reported behavior as bioactive volatiles, i.e., VOCs which have a role in bacteria adaptation to an environment and inter-species signaling [39]. Therefore, bio-AgNPs may elicit the production of biologically active VOCs, enabling bacteria to better adapt to the modified medium. However, VOCs with such properties are generally present at lower concentrations of nanoparticles (C1, or even C2), evidencing the predominance of deleterious effects of nanoparticles over the ability of bacteria to customize their metabolism according to the environment.

Spearman correlation coefficients (rho) were calculated for each of the detected VOCs, aiming to point out the compounds that, in a significant manner, had their responses greatly modulated by the applied concentrations of agent. General guidelines for the interpretation of presented coefficients is described by the literature on statistical methods [64]. A very strong positive correlation was found between the compound area and the concentration level of bio-AgNPs for 2-undecenal in *E. coli* (rho = 0.97, *p =* 7.88 × 10^−8^), *E. faecalis* (rho = 0.89, *p =* 1.03 × 10^−4^) and *K. pneumoniae* (rho = 0.93, *p =* 8.79 × 10^−6^); 2-decenal in *E. faecalis* (rho = 0.86, *p =* 3.11 × 10^−4^); nonanoic acid in *K. pneumoniae* (rho = 0.83, *p =* 7.74 × 10^−4^) and 2,4-decadienal in *P. mirabilis* (rho = 0.85, *p =* 5.15 × 10^−4^). A strong positive correlation was identified for 2,6-dimethylnonane in *E. coli* (rho = 0.71, *p =* 8.36 × 10^−3^) and 2-ethyl-5-methyl-pyrazine in *E. faecalis* (rho = 0.80, *p =* 1.84 × 10^−3^). These are VOCs which were proportionally elevated in response to increasing amounts of nanoparticles. On the other hand, very strong negative correlation was detected for 2-nonanone in *E. coli* (rho = −0.84, *p =* 6.11 × 10^−4^) and *P. mirabilis* (rho = −0.82, *p =* 1.28 × 10^−3^). A strong negative correlation was presented by propyl propanoate in *E. faecalis* (rho = −0.80, *p =* 1.62 × 10^−3^), *K. pneumoniae* (rho = −0.76, *p =* 4.10 × 10^−3^); 2-octanol (rho = −0.74, *p =* 5.77 × 10^−3^) in *E. faecalis*; in *K. pneumoniae*, allyl thioacetate (rho = −0.71, *p =* 8.42 × 10^−3^), 2-nonanone (rho = −0.75, 4.30 × 10^−3^) and S-methyl propanethioate (rho = −0.79, *p =* 1.92 × 10^−3^); in *P. mirabilis*, methyl isopropyl disulfide (rho = −0.73, *p =* 6.87 × 10^−3^), S-methyl-3-methylbutanethioate (rho = −0.73, *p =* 6.57 × 10^−3^), methyl ethyl disulfide (rho = −0.74, *p =* 5 × 10^−3^) and 2-butanol (rho = −0.78, *p =* 2.71 × 10^−3^).

The antimicrobial mechanism of action of AgNPs implies in the interaction of nanoparticles with bacterial biomolecules, eventually promoting their degradation—directly or indirectly—due to the release of ROS in the cell. As mentioned, aldehydes and fatty acids that elevated their levels as progressively greater concentrations of agent were applied can be pondered as by-products of lipid peroxidation. The crescent responses of 2,6-dimethylnonane may be associated with similar processes targeting branched lipids. The occurrence of branched fatty acids among bacterial lipids is common and has been associated to functions related to regulation of membrane fluidity. The ratio of *iso*:*anteiso* branched fatty acids also seems to be involved in bacteria adaptation to pH and temperature [65]. The decarboxylation of fatty acids can give rise to corresponding alkanes [18]. Apart from this, alkanes can be obtained in the course of peroxidation of a fatty acid followed by its cleavage and posterior reduction [66]. In this sense, elevation of branched species can be linked to the degradation of constituting lipids. Similar hypothesis can be drawn regarding the previously mentioned appearing/augmentation of 2,6,7-trimethyldodecane and nonane, observed at C2.

Interestingly, most of the compounds negatively regulated were VSCs, demonstrating that the catabolism of S-containing amino acids appears largely impaired for *K. pneumoniae* and *P. mirabilis*. The biogenesis of VSCs, including S-methyl thioesters, is often related to methionine and cysteine derivatives, and part of these volatiles are assigned as bioactive molecules [18]. Such a fact can be related to the affinity of AgNPs for sulfur-containing components, which may result in the removal of VSCs-precursor molecules from the biological environment through the formation of inorganic sulfides [67,68]. Another hypothesis is linked to the possible inhibition of enzymes responsible for the biotransformation of S-amino acids. Moreover, 2-nonanone configures as negatively modulated for all studied bacterial species. This compound can appear to be typically formed during the oxidation of β-keto acids, therefore being connected with the catabolism of lipids [69]. The reduction of methyl ketones can lead to the formation of secondary alcohols such as 2-octanol [69], which also was found decreasing while concentrations of bio-AgNPs increased.

### 2.6. Ascertainment of Affected Bacterial Pathways

**Figure 7:**
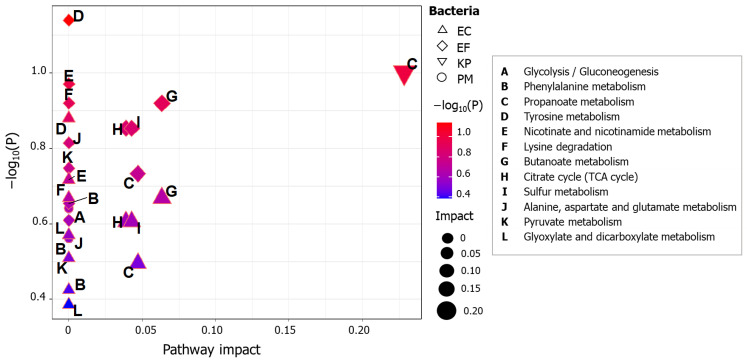
Pathway topology analysis based on KEGG-described paths. EC = *E. coli*, EF = *E. faecalis*, KP = *K. pneumoniae*, PM = *P. mirabilis*.

MetaboAnalyst is an online platform that offers several tools for data analysis on metabolomics. Pathway analysis (PA) allows us to determine the main metabolic paths affected by a studied event, using as input a list of metabolites that were found significantly altered. The entries are then related to a repository containing pathways already fully described by the literature, for a given organism (in this case, KEGG pathway database resource). MetaboAnalyst PA module reports measurements of pathway impact and node importance. The importance can be expressed in terms of number of shortest paths passing through a specific node (betweenness centrality), in this manner, compounds with potential to regulate a greater number of processes are considered more relevant. In addition, pathway impact value is given by the sum of the importance of the matched entries normalized by the sum of the importance of the total number of metabolites contained in a referred pathway [70]. Figure 7 presents a weighted plot that uses as input *p*-values and pathway impact calculated by MetaboAnalyst PA tool. Most of the discriminating metabolites that matched to the database source were those detected in *E. coli* and *E. faecalis* species. Tricarboxylic acid (TCA) cycle, propanoate metabolism and sulfur metabolism configured as the most affected pathways in *E. coli*. Similar results were verified for *E. faecalis* (with effect on butanoate instead of propanoate metabolism), suggesting that bio-AgNPs action was analogous for these species. For *K. pneumoniae*, propanoate metabolism was reported as notably disturbed. *P. mirabilis* glycolysis and phenylalanine metabolism appear influenced by bio-AgNPs supplementation; however, the impact calculated for these pathways was low. In general, this PA method denoted that main dysregulated processes present to be those regarding releasing of energy for the cell and the processing of amino acids.

**Figure 8:**
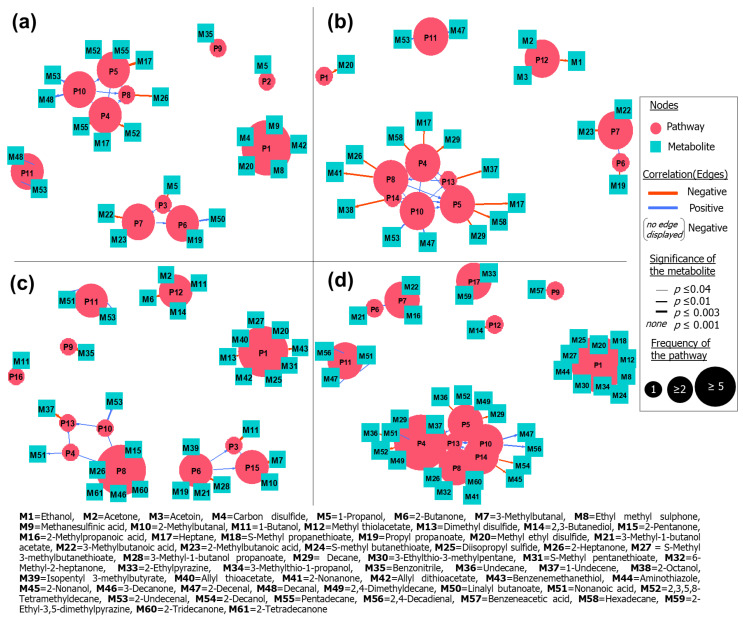
Pathway network analysis presenting the relations between most relevant metabolites (squares) and likely associated bacterial processes (circles) for (**a**) *E. coli*, (**b**) *E. faecalis*, (**c**) *K. pneumoniae* and (**d**) *P. mirabilis*. P1 = metabolism of S-containing amino acids, P2 = propanoate metabolism, P3 = Ehrlich pathway—reduction of keto acids, P4 = fatty acids pathway—fatty acid biosynthesis, P5 = fatty acids pathway—decarbonylation of fatty aldehydes, P6 = Ehrlich pathway—esterification of fusel alcohols, P7 = Ehrlich pathway—oxidation of fusel aldehydes, P8 = fatty acids pathway—decarboxylation of keto fatty acids, P9 = aldoxime–nitrile pathway—phenylalanine metabolism, P10 = fatty acids pathway—reduction of fatty acyl-ACP, P11 = lipid peroxidation, P12 = pyruvate fermentation, P13 = fatty acids pathway—decarboxylation of fatty acids, P14 = fatty acids pathway—reduction of methyl ketones, P15 = Ehrlich pathway—decarboxylation of keto acids, P16 = butanoate metabolism, P17 = amino acids metabolism—tryptophan metabolism.

Nevertheless, it is important to highlight that such a PA method is limited to the data available in the used repository that are given for model organisms, and it comprehends solely mechanisms that have been fully elucidated and registered. More specific processes take place in the bacterial metabolism, reflecting on the observed variety of compounds. In this sense, another approach to study affected pathways was applied. For this purpose, we considered compounds that appeared significantly altered when the control and all bio-AgNPs-treated cultures were compared—it means, compounds that configured as consistently altered in treated samples, regardless of applied concentration of agent. The approach consisted of assigning the found discriminating compounds to general metabolic processes reported by the previous literature. Figure 8 is a network exhibiting the connections between volatiles and the metabolic mechanisms that possibly originate them, for each of the investigated species. Mechanisms belonging to the same path appear interconnected. The extension of the influence on a pathway can be inferred from the frequency that their addressed metabolites appear altered. In this manner, pathway processes with greater frequency values are those that were more disturbed by the action of added nanoparticles.

The metabolism of S-containing amino acids appears as the most downregulated process in *E. coli*, *K. pneumoniae* and *P. mirabilis*. Regarding the fatty acids’ pathway, only the reduction of fatty acyl-ACP, resulting in fatty aldehydes, appeared as possibly enhanced mechanism. However, metabolites of this latter path overlap with lipid peroxidation process. Negative modulation of the aldoxime–nitrile pathway was evidenced for the studied bacteria, with exception of *E. faecalis*. This route provides source of carbon, nitrogen and, eventually, bioactive molecules. Generated nitriles can be transformed into the corresponding carboxylic acid and ammonia [71]. In this context, benzonitrile is the metabolite provided from the processing of phenylalanine. Decreased levels of this VOC indicate the enzymatic inhibition of the first step of the referred path, which comprises amino acids conversion to nitrile by action of cytochrome p450 [71]. The esterification of fusel alcohols also presented itself as boosted in *E. coli*. The overall increasing in the production of esters, which are final product of Ehrlich pathway, can be connected with an amplification of the catabolism of amino acids assimilated by this path—in this particular strain, it is possible that such mechanism is being used by cell as an alternative process to promote energy-efficient NADH regeneration, due to the detriment of respiration as a main energetic source. In *E. faecalis*, as for *E. coli*, the majority of altered metabolites belongs to the biochemical path of lipids. However, in this case, fermentation processes also configure as considerably hampered. Regarding *K. pneumoniae*, the decarboxylation of keto fatty acids appears reduced, factor evidenced by the suppressed emission of several methyl ketones. Early steps of fatty acids path were not so impacted as it was observed for other bacterial species. Depletion of fermentation processes is also expressive. Apart from the examination of qualitative VOC profiles, the tracking of impaired biochemical routes also suggests that *P. mirabilis* was the bacterium more susceptible to the presence of bio-AgNPs. For this species, nanoparticles caused decreased response of numerous S-containing metabolites and great impairment of fatty acids routes (mostly affecting biosynthesis and β-carboxylation steps). Moreover, *P. mirabilis* displayed increasing trends for three metabolites possibly descendant from lipid peroxidation. Depletion of pyrazines emerge as an indicator of restricted catabolism of tryptophan. In agreement with the general remarks provided by this analysis, an in silico approach aiming to predict the interaction between AgNPs and proteins revealed that inhibition is expected for enzymes involved in the metabolism of several amino acids, glycolysis, TCA cycle and ions transportation, evidencing the interruption of essential cellular activities and the participation of oxidative processes [72].

A greater bacterial resistance against bio-AgNPs can be inferred from VOC patterns of *E. faecalis*, once these were the less affected in terms of conservation of the original volatilome. Indeed, previous studies on molecular profiles of Gram-positive and Gram-negative bacteria, showed that this first group presented to be superior is terms of resistance against bio-AgNPs, especially when a longer incubation time with the agent is considered. Protein patterns acquired by MALDI–TOF–MS technique for *S. saccharolyticus* were resumed after 12 h of incubation with bio-AgNPs, while Gram-negative species suffered more extensive alteration of their original molecular profiles [21]. The negative charges of lipopolysaccharides composing the outer membrane of Gram-negative bacteria probably exert attraction on the residual positive charges from nanoparticles. The thick layer of peptidoglycan, composed by crosslinked chains of polysaccharides, confers improved rigidity to the structure of bacterial cell, resulting in a greater obstacle for the penetration of AgNPs and creating lower availability of anchoring sites for nanoparticles [73]. Among Gram-negative bacteria, *E. coli* configured as the one with less disturbed metabolism.

Once the antimicrobial action of metal nanoparticles possibly relies on their simultaneous interaction with bacterial cell components, the inhibition of resistance mechanisms could be supposed [6]. However, bacterial resistance to nanosilver has been documented and may involve genetic and phenotypic alternations. A mechanism based in the production of flagellin protein in *E. coli* 013, *P. aeruginosa* CCM 3955 and *E. coli* CCM 3954 repeatedly exposed to AgNPs effectively induced nanoparticles aggregation [74]. Another study observed that nanosilver led to selective adaptation of *Bacillus* sp., causing these to dominate a local model microbiota where *E. coli* was previously predominant; the mechanism is possibly elicited by *Bacillus* sp. resistance to cellular conditions of oxidative stress [75]. Naïve *E. coli* strain was evolved in a AgNP environment and developed resistance probably linked to the occurrence of accumulating mutations retained in the treatment group [76]. The application of AgNPs in the soil and plant environment appeared to shift the pattern of antibiotic resistance genes. The noticed augmentation of relative abundance of efflux pumps genes indicates that AgNPs may contribute to the propagation of antibiotic resistance via co-selection mechanism [77].

On the other hand, AgNPs tested against multidrug-resistant *K. pneumoniae* MGH78578 proved to be effective by causing a triclosan-like bactericidal action, accompanied by the inhibition of fatty acid biosynthesis and notable triggering of oxidative stress [78]—effects also pointed out by the present study. Mechanistic model of action of AgNPs on *P. mirabilis* revealed that agent effectiveness was deeply related to the release of free radicals, what induced lethal activity by deprivation of nutrients and signal supply [79]. However, *P. mirabilis* SCDR1 isolated from a diabetic ulcer presented resistance against commercial Nanosilver products. Pathogenomics analysis showed that this strain carries several mechanisms related to biofilm formation, mobility, active transport and other detoxification methods, which may induce the observed resistance [80].

**Figure 9:**
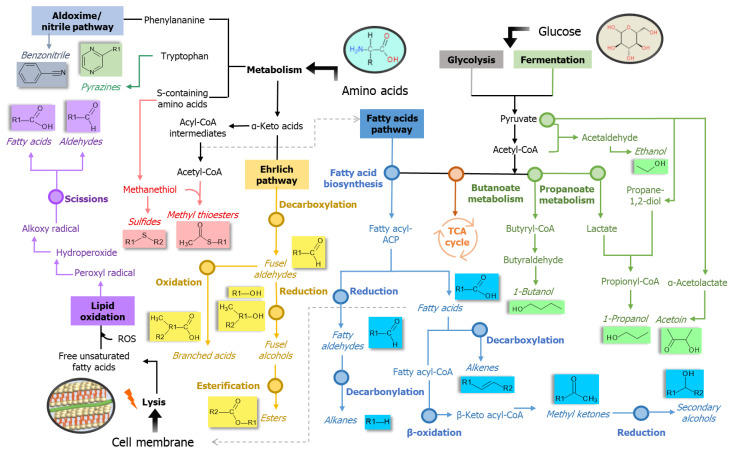
Scheme presenting interconnected bacterial pathways possibly affected by the supplementation of the culture medium with bio-AgNPs. CoA = coenzyme A, TCA = tricarboxylic acid, ACP = acyl carrier protein, S = sulfur, ROS = reactive oxygen species.

A schematic representation of pathways potentially impacted by the presence of bio-AgNPs is shown in Figure 9. The scheme contains the volatile final products of each cellular process represented. It was denoted that interference on each of the bacterial paths is dependent on agent concentration. Overall, at lowest concentration there is a stimulation in the production of bioactive compounds, which probably play a role in supporting microorganism adjustment to the medium. Besides that, fermentation pathways appear enhanced, implying yet a partial inhibition of respiratory chain functions. The addition of the two greater levels of bio-AgNPs caused noticeable loss in the variety of assessed volatilomes. The noticed interruption in the biosynthesis of VSCs can be a reflex of nanoparticles binding to precursors molecules and the inhibition of carbon–sulfur lyase enzymes responsible for cleaving S-containing amino acids [81]. β-oxidation cycle can be interpreted as an option for the origination of energy in view of deficient respiration after AgNPs action [82]. In a first step, it is possible that the positive modulation of certain fatty acids products, like alkanes, may be an indicative of such metabolic operation. Nevertheless, efficient means for bacteria survival appear to be undermined when the greatest concentration of nanoparticles is applied. In this case in point, it is verified that there was a sudden decay in the production of several typical bacterial metabolites and a magnified emission of aldehydes.

The proposed experimental approach enabled metabolites sampling, pre-concentration and introduction in the analytical system in a single step, with minimal sample handling. As drawback, only the volatile/semi-volatile portion of the produced metabolites was assessed. Other omics approaches may involve the analysis of bacteria genetic material or the screening for non-volatile compounds, such as amino acids, proteins and lipids. Each of these experimental designs offers a different fraction of information regarding the metabolism of the investigated biological system. Such knowledge combined can offer a completer and more detailed panel of affected metabolic processes. 

## 3. Material and Methods

### 3.1. Sample Preparation

#### 3.1.1. Bacteria Isolation and Identification

Bacterial strains used in this study (*Escherichia coli* MB11464_1, *Enterococcus faecalis* DSM 20409 DSM, *Klebsiella pneumoniae* 9295_1 CHB and *Proteus mirabilis* (PX) 22086112 MLD) were isolated from the wound culture collected by the specialist nurse, applying the Levine technique, according to the local guidelines. A flocked swab (ESwab Collection System—Copan, Murrieta, CA, USA) was used to collect the samples from a clean viable tissue area (1 cm^2^), avoiding the necrotic, non-viable tissue or slough. Then, the swabs were transferred to liquid Amies medium (ESwab Preservation System, Copan) and transported to the laboratory. Each swab sample was spread directly onto trypticase soy agar (TSA medium—Sigma-Aldrich, Steinheim, Germany), and the plates were incubated at 37 °C for 24 h. Isolation of bacterial cells was performed by serial dilution method, using peptone water (Sigma-Aldrich, Steinheim, Germany). An aliquot of prepared dilution was cultured on TSA and incubated in aerobic conditions at 37 °C for 24 h. Based on the shape, size and color, the colonies were selected and streaked on separate plates. The separated colonies were identified by MALDI–TOF/TOF–MS system (matrix-assisted laser desorption/ionization time-of-flight mass spectrometry—ultrafleXtreme™, Bruker Daltonics, Bremen, Germany), using MALDI Biotyper^®^ Compass platform (Bruker Daltonik GmbH, Bremen, Germany) based on Raw and Main Spectra (MSP).

#### 3.1.2. Minimal Inhibitory Concentrations (MIC)

Silver nanocomposites used in this study were synthesized by using *Lactobacillus curvatus* DSM20496DSM as a source [83]. Then, the obtained nanocomposites were characterized (unpublished data). For the evaluation of MIC of the biosynthesized agent, seven concentrations (100, 50, 25, 12.5, 6.25, 3.125 and 1.56 µg mL^−1^) of silver nanocomposites were investigated against the selected pathogens (*Escherichia coli* MB11464_1 CHB, *Enterococcus faecalis* DSM 20409 DSM, *Klebsiella pneumoniae* 9295_1 CHB and *Proteus mirabilis* (PX) 22086112 MLD). Solutions at aforementioned concentrations were mixed in a 96-well round-bottom plate (Sigma Aldrich, Poznań, Poland) with the cultured bacterial cells (1 × 10^6^ CFU/mL), in a ratio of 1:1. In this procedure, three replicates were prepared per assay. In order to determine the MIC value, 12 μL of resazurin-based in vitro toxicology assay kit (Sigma-Aldrich, St. Louis, MO, USA) was added to each well. Next, the 96-well round bottom plates were incubated, under continuous stirring, for 24 h, at 37 °C, in the dark. Based on the metabolic activity of living cells expressed by the changed of the color from blue to pink, the MIC values were determined. Untreated bacterial cells were used as the control. The control samples were prepared at a ratio of 1:1 of pure medium and cultured bacterial cells (1 × 10^6^ CFU/mL). These assays were all performed in Mueller-Hinton broth medium (MH) (Sigma Aldrich, Poznań, Poland), according to the guidelines of the Clinical and Laboratory Standards Institute (CLSI).

#### 3.1.3. Sample Preparation for HS-SPME/GC–MS Analysis

Headspace screw top 20 mL clear vials and magnetic polytetrafluoroethylene (PTFE)/silicon screw caps (18 mm thread) were purchased from Agilent (Santa Clara, CA, USA). Prior to experiments, culture medium, nanoparticles and vials were autoclaved at 121 °C for sterilization. Vials’ screw caps were sterilized by being evenly exposed to ultraviolet radiation for 1 h. In order to verify the influence of different concentrations of silver nanoparticles on VOC profiles of selected pathogens, three concentrations of the prepared agent were selected for examination: the MIC value and two other concentrations higher than this one. Four milliliters of MH broth was transferred to a vial. Then, the content was inoculated with a bacterial suspension (already cultured bacteria, at 37 °C for 24 h) of the studied pathogens, prepared in the same medium. Before application of bio-AgNPs, optical density of the inoculum was consistently verified, being approximately 1 McFarland in all cases. Immediately, the nanoparticles were added to obtain the desired concentrations. Prepared sample vials (three replicates per assay) were placed in an incubator at 37 °C to promote appropriate bacterial growth, being taken out for analysis immediately after 24 h. Several blank samples (untreated bacterial cells, pure medium and medium treated with used concentrations of agent) were prepared in order to be paired with all respective positive assays.

### 3.2. HS-SPME/GC–MS Analysis

In order to favor thermodynamic equilibrium, samples were pre-incubated at 37 °C, for 30 min. VOCs extraction was conducted at 37 °C for 10 min, using a 65 µm polydimethylsiloxane (PDMS)/divinylbenzene (DVB) fiber (Supelco, Bellefonte, PA, USA). Then, the loaded SPME fiber was desorbed into GC inlet port for 2 min. All GC–MS experiments were done in triplicate. Carrier gas (helium 6.0) flow rate was set as 2.2 mL min^−1^, and inlet temperature was set at 250 °C. The oven temperature program was as follows: the initial temperature of 40 °C was kept for 4 min, then ramped at 100, 220 (held for 7 min) and 250 °C, at respective rates of 10, 15 and 20 °C min ^−1^. The oven was maintained at this last temperature for 5 min. Spectra acquisition was performed within the range of m/z 25–300, using electron ionization (EI) at 70 eV. Both ion source and the transfer line temperatures were set as 220 °C, while the quadrupole analyzer was kept at 150 °C.

The GC–MS analyses were carried out by using an Agilent 6890A gas chromatograph coupled to an Agilent 5975 Inert XL MSD mass spectrometer (both from Agilent Technologies, Santa Clara, CA, USA), associated with an autosampler MPS2 (Gerstel, Sursee, Switzerland). The system was equipped with a DB-624 UI 60 m × 0.25 mm × 1.40 µm column (Agilent, Palo Alto, CA, USA). Compound identification was processed by searching the obtained mass spectrum in the NIST11 mass spectral library. The criterion for peak detection was a signal-to-noise of at least 3, and peak integration was done manually. Spectrum search encompassed baseline subtraction and averaging over a peak. Forward and reverse match quality of at least 750/1000 was considered as the lower match threshold. Peaks detected in the corresponding blanks were deleted from the total dataset, for the obtainment of signals attributed solely to bacterial activity.

### 3.3. Statistics and Data Analysis Approaches

The following methods were processed in R environment, using RStudio v. 1.1.463 console (RStudio, PBC, Boston, MA, USA). PCA was conducted by using “prcomp” function, in order to visualize general trends of the data regarding VOC bacterial profiles. HCA associated with heatmap was generated by applying “pheatmap” package, cluster distance was measured by using Pearson correlation coefficient and Ward’s minimum variance method (“ward.D2”) was the applied clustering criterion. For both PCA and HCA, data scaling was performed prior analysis (by subtracting variable’s mean and dividing it by the standard deviation). Venn diagram and networks aiming to represent the volatiles coincident among the bacterial strain groups were prepared, using “VennDiagram” and “sna” packages, respectively. General plots were produced with the aid of “ggpubr” and “ggplot2” packages. Dot plots aimed to reveal the trend presented by the detected volatiles after the application of bio-AgNPs in relation to the corresponding unstressed strains, in terms of binary logarithm of compound fold-change. Weighted plot used as input the pathway impact and significance (in terms of -log_10_ p) calculated by the pathway analysis tool of the web server MetaboAnalyst 4.0 [84]. The following parameters were employed: over-representation analysis = hypergeometric test; pathway topology analysis = relative-betweenness centrality; model organisms = *Escherichia coli* K-12 MG1655 and *Klebsiella pneumoniae* MGH 78578 (serotype K52)—such an online tool uses pathways that are described and stored at the Kyoto Encyclopedia of Genes and Genomes (KEGG database) [85]. Ultimately, using the “igraph” package, we performed a network analysis to show links between the relevant metabolites (fold-change in relation to unstressed culture with *p* < 0.05) and possibly related biochemical processes. Spearman correlation analysis was conducted to verify the reproducibility of VOC profiles obtained from analyzed replicates—coefficients ≥0.8 were obtained for replicates belonging to the same assay; the Mann–Whitney U test was performed to point out discriminating features, considering *p* < 0.05 as the relevance criterion. The latter procedures were conducted by using IBM SPSS Statistics v. 24 (IBM Corp., Armonk, NY, USA).

## 4. Conclusions

A comprehensive investigation of VOCs extracted from the headspace of cultures of *E. coli*, *E. faecalis*, *K. pneumoniae* and *P. mirabilis* treated with bio-AgNPs was carried out. Firstly, the profile of released volatile compounds appear to be characteristic for each of the studied pathogens, serving as a fingerprint of individual metabolic systems and the with potential to be applied for discrimination between bacterial species. Used data analysis methods also allowed us to unravel the biochemical meaning of altered patterns of VOCs. Each of the tested concentrations of agents induced different metabolic responses on bacteria. The application of nanoparticles at 12.5 μg mL^−1^ demonstrated that it triggers the biosynthesis of several bioactive volatiles, which appear to be connected with signaling, enhancement of virulence, biofilm formation and bacterial resistance. Besides that, alternative processes of energy obtaining are elicited—fermentation and β-oxidation of fatty acids. Increasing concentrations of nanoparticles evidenced interaction between ROS and fatty acids which compose the bacterial cell membrane, indicating the progressive damage of plasma membrane, and, therefore, successful incorporation of bio-AgNPs. Such a process was accompanied by the inhibition of pathways mainly related to the metabolism of amino acids and fatty acid biosynthesis and catabolism, resulting in the abrupt decrease in the variety of produced volatile metabolites. VOC profiles from supplemented cultures of *E. faecalis* were the most preserved in comparison to the untreated ones, suggesting their lower susceptibility to the bio-AgNPs. On the other side, it is estimated that *P. mirabilis* lost 80% of its metabolic diversity, while the response of three potential oxidative stress indicators (2-decenal, 2,4-decadienal and nonanoic acid) increased together with the added concentrations of agent. Evaluation of VOCs emitted by bacterial cultures proved to be an efficient tool to monitor metabolic changes and molecular reactions induced by antimicrobial agents, enabling us to elucidate the particularities of its action over different bacterial species. In addition, such an approach can be useful to foresee and track resistance mechanisms or aspects of agent effectiveness.
